# Recent Advances in Targeted Drug Delivery Strategy for Enhancing Oncotherapy

**DOI:** 10.3390/pharmaceutics15092233

**Published:** 2023-08-29

**Authors:** Jianmin Li, Qingluo Wang, Guoyu Xia, Nigela Adilijiang, Ying Li, Zhenqing Hou, Zhongxiong Fan, Jinyao Li

**Affiliations:** 1 College of Life Science and Technology & Institute of Materia Medica, Xinjiang University, Urumqi 830017, China; 107552201047@stu.xju.edu.cn (J.L.); 107552203673@stu.xju.edu.cn (Q.W.); xgy1823@stu.xju.edu.cn (G.X.); nigara@stu.xju.edu.cn (N.A.); 2 Xiamen Key Laboratory of Traditional Chinese Bio-Engineering, Xiamen Medical College, Xiamen 361021, China; 3 College of Materials, Xiamen University, Xiamen 361002, China; houzhenqing@xmu.edu.cn

**Keywords:** nanocarriers, targeted delivery, active targeting, passive targeting, oncotherapy

## Abstract

Targeted drug delivery is a precise and effective strategy in oncotherapy that can accurately deliver drugs to tumor cells or tissues to enhance their therapeutic effect and, meanwhile, weaken their undesirable side effects on normal cells or tissues. In this research field, a large number of researchers have achieved significant breakthroughs and advances in oncotherapy. Typically, nanocarriers as a promising drug delivery strategy can effectively deliver drugs to the tumor site through enhanced permeability and retention (EPR) effect-mediated passive targeting and various types of receptor-mediated active targeting, respectively. Herein, we review recent targeted drug delivery strategies and technologies for enhancing oncotherapy. In addition, we also review two mainstream drug delivery strategies, passive and active targeting, based on various nanocarriers for enhancing tumor therapy. Meanwhile, a comparison and combination of passive and active targeting are also carried out. Furthermore, we discuss the associated challenges of passive and active targeted drug delivery strategies and the prospects for further study.

## 1. Introduction

Tumors, as a major global public health issue, pose a serious threat to human health [1]. Recently, traditional therapeutic strategies (e.g., surgery, chemotherapy, and radiotherapy) have remained the mainstream oncotherapy strategies. However, there are many limitations to these therapeutic strategies. For example, although surgery can directly remove the visible tumor tissue, it cannot do anything about the tumor’s spread and metastatic sites. In addition, chemotherapy can kill tumor cells, but it lacks specificity and indiscriminately kills normal cells or tissues [2]. Radiotherapy can result in different degrees of damage toward normal tissues [3]. Therefore, researchers are widely exploring various therapeutic strategies to reduce its undesirable side effects toward normal cells or tissues. In addition, it should be pointed out that more precise and effective targeted drug delivery strategies that have emerged are promising therapeutic strategies for various diseases, especially tumors.

Targeted delivery strategies can precisely and effectively deliver most drugs to tumor cells or tissues instead of normal cells or tissues [4]. Such delivery strategies can be achieved using nanotechnology. Typically, nanoparticle-based drug delivery has attracted increasing attention because nanoparticles can accumulate at tumor sites through the EPR effect [5]. A main component of targeted drug delivery systems is the ‘targeting fraction’, which can specifically bind with certain moieties or receptors at the target site. Moreover, targeted drug delivery can achieve the goal of personalized therapy due to its low drug dosage, high efficacy, and few side effects. As is well known, targeted nanocarriers can effectively improve the bioavailability and efficacy of drugs via various targeting mechanisms. For example, nanocarriers can change the pharmacokinetics and tissue distribution of drugs and enhance the intracellular uptake of drugs. Compared with other delivery strategies, the advantages of nanocarrier-based targeted drug delivery can be summarized as follows: targeted drug delivery can significantly improve the drugs’ therapeutic effects and reduce their side effects by selectively delivering drugs to tumor sites. Furthermore, the application of nanotechnology can improve the stability and bioavailability of drugs, thereby extending their therapeutic effect and reducing the frequency of administration and dosage of drugs. Recently, nanocarrier-based targeted drug delivery mainly includes passive targeting and active targeting.

**Figure 1:**
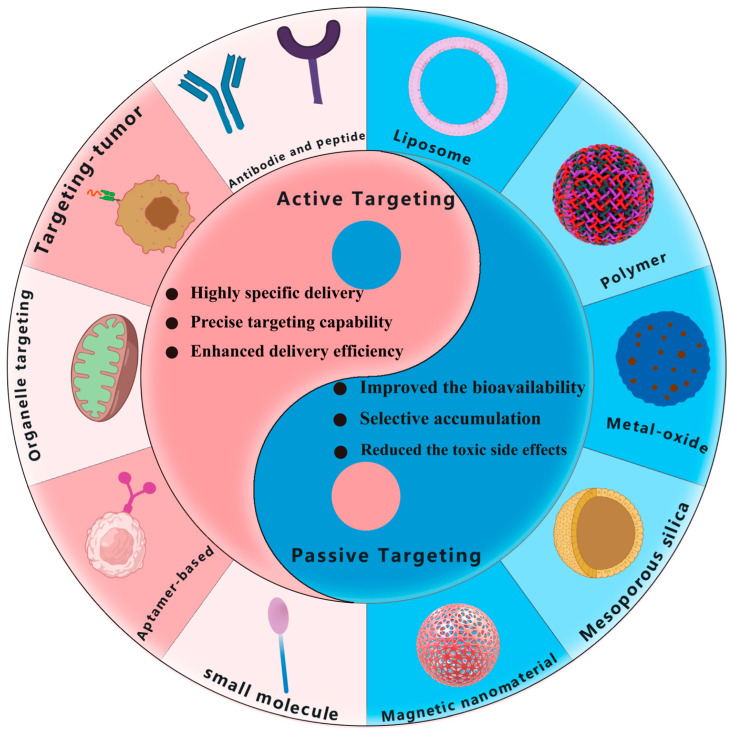
Schematic illustration of nanocarrier-based active and passive targeting drug delivery systems.

Nanocarriers can be regarded as a popular drug carrier for targeted drug delivery to improve the bioavailability, biodistribution, and accumulation of drugs. In addition, to reduce immunogenicity, nanocarriers can not only modify via controlled chemosynthesis but can also load one or more drugs. Nanocarriers can enhance the targeting effect on tumor cells through surface modifications to precisely deliver drugs to tumor tissue. Such a precise delivery strategy can increase the tumor accumulation of drugs, thereby reducing their toxic side effects on normal cells or tissues [6]. For example, the physicochemical properties and drug release behaviors can be managed by changing the particle size, shape, and surface [7]. In addition, nanocarriers can specifically interact with biomolecules, thereby enhancing their stability and circulation time. Furthermore, nanocarriers can integrate multiple drugs and therapeutic approaches for synergistic oncotherapy. Recently, the various developed nanocarrier-based targeted drug delivery strategies have mainly included the introduction of targeting molecules (antibodies, peptides, aptamers, small molecules, etc.) and delivery vehicles (liposomes, polymers, metal oxides, silica, etc.) [8]. The design and optimization of these targeting molecules and delivery vehicles enable a strong interaction with tumor cells and improve the delivery efficiency and specificity of drugs. In addition, nanocarrier-based targeted drug delivery can enhance drug accumulation in tumor tissue and reduce drug distribution in normal tissue [9,10]. Although significant progress has been made in targeted drug delivery in oncotherapy, many challenges and limitations, such as the complexity of tumor structure, the ambiguous biosafety of delivery systems, as well as the rapid metabolism and clearance of drugs, still remain. Therefore, further research is still necessary to promote the development and clinical application of targeted drug delivery technologies in efficient oncotherapy [11]. In light of the above considerations, as shown in Figure 1, this paper will review the recent advances in and applications of targeted drug delivery in oncotherapy and clarify the mechanisms of drug delivery via different nanocarriers with passive and active targeting effects, through which we expect to provide references and insights to stimulate further development in the field of targeted drug delivery and provide multiple strategies for oncotherapy.

## 2. Passive Targeting Targeted of Drug Delivery

**Figure 2:**
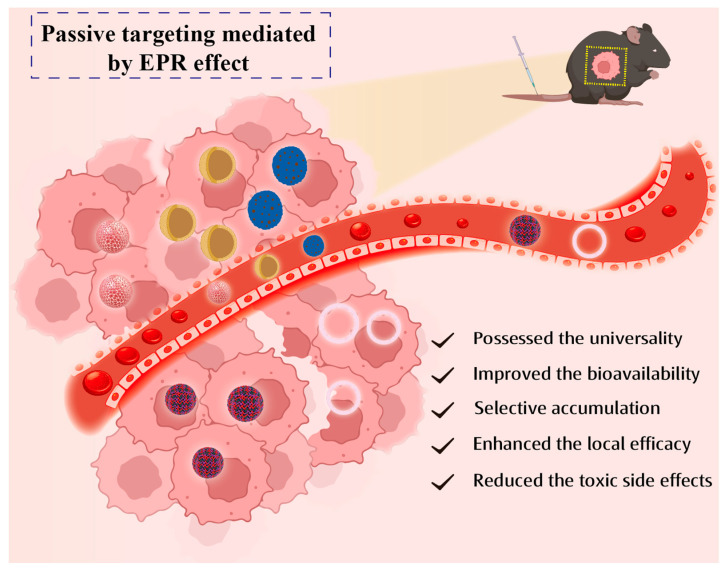
Mechanism and advantages of passive targeting.

The mechanism of passive targeting is (see Figure 2) that nanocarriers can deliver drugs into the tumor mesenchyme or cells through the interstices of tumor capillary pores via passive diffusion or convection [12]. Convection is the movement of molecules within a fluid. When the net filtration rate reaches zero, convection becomes the predominant mechanism for transporting the majority of macromolecules through the vascular pore spaces. In contrast, low-molecular-weight compounds, such as oxygen, are primarily transported by diffusion. According to a concentration gradient, diffusion can be described as the mechanism by which molecules are transported across the cell membrane without the expenditure of cellular energy. However, the limited convection through the tumor mesenchyme caused by elevated interstitial pressure makes diffusion the main mode of drug transportation. Passive strategy is typically <150 nm of particle-mediated EPR effects (a physiological phenomenon unique to solid tumors), which is characterized by a greater permeability of capillaries surrounding solid tumors compared to normal tissue and the absence of functional lymphatic vessels. This physiological phenomenon allows nanoparticles within the blood (size: ca. 250 nm) to efficiently reach the tumor site through convection or passive diffusion through the tumor capillary pores easily, thereby accumulating in tumor tissues and gradually penetrating into the tumor mesenchyme and intracellular compartments upon time elapsing. This phenomenon is the theoretical foundation for the passive targeting of nanodrugs to various tumors [13,14]. A large number of authoritative research has revealed that the classical EPR effect applies to nearly all fast-growing solid tumors [15]. The passive targeting drug delivery systems mainly include liposomes, polymeric nanoparticles, metal oxide nanoparticles, silicon dioxide nanoparticles, etc.

### 2.1. Liposomes

A large number of nanocarriers with the passive targeting effect have been developed upon the rapid development of nanotechnology. For example, liposomes, as a well-established drug vesicle, have been widely employed in drug delivery. On one hand, lipids play a crucial role as a fundamental component of cell membranes. Additionally, lipids are highly biocompatible with each other, and the utilization of liposomes as nanocarriers does not consider their biodegradation [16]. On the other hand, lipids can be wrapped on the surface of nanodrugs. Therefore, whether hydrophilic or hydrophobic, inorganic or polymeric materials can form the supported lipid monolayers or bilayers [17,18,19], where the fluid lipid surfaces also facilitate the attachment of targeted ligands, thereby achieving a good combination of nanoparticles with active targeting and passive targeting effects. The biophysical properties of lipid membranes play a significant influence on drug delivery. Furthermore, the various properties such as lipid structural domain formation, mobility, multivalent binding, leakage, and fusion, are all used for drug delivery. Liposomes are softer and more easily deformed than inorganic delivery carriers. Fluidic membrane is beneficial for the dynamic recombination of ligands. Therefore, these properties confer excellent passive and active tumor-targeted capabilities to liposomes.

**Figure 3:**
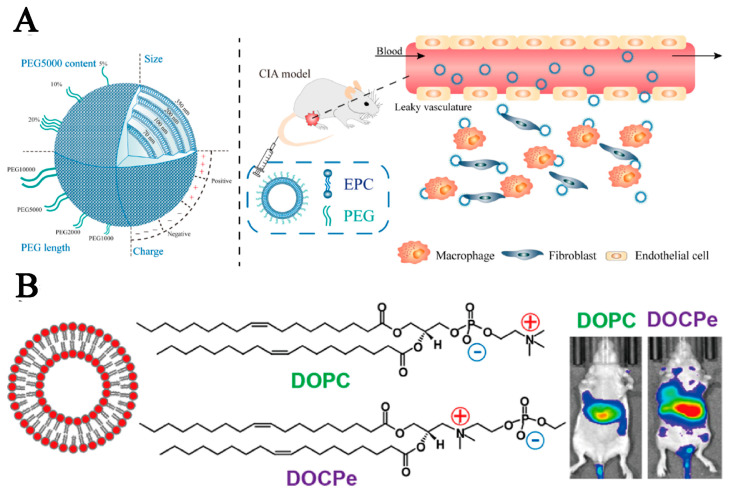
The application of liposomes obtained using different assembly methods to reduce the drug leakage of lipid-containing nanoparticles and prolong the blood circulation time for enhancing their passive tumor-targeted ability. (**A**) Reproduced with permission from Ref. [23]. Copyright 2019, American Chemical Society. (**B**) Reproduced with permission from Ref. [25]. Copyright 2017, American Chemical Society.

The passive targeting of liposomes involves the accumulation of liposomes loaded with drugs in tumors based on EPR effects, whereas active targeting relies on the binding of liposomes to specific ligands [20]. The size of liposomes, as an important parameter, is associated with blood circulation time, leakage vascular extravasation, and macrophage uptake. In addition, its size effect on drug delivery has been studied. In general, the decrease in liposome size will reduce their cellular uptake via the monocyte/phagocyte system and increases the blood circulation time. Liposomes at ca. 100 nm are most commonly used to prolong blood circulation and improve passive targeting [21,22]. A study in rheumatoid arthritis also showed that liposomes based on egg yolk lecithin at 100 nm had a prolonged blood circulation time (12.85 h) than that at 70 nm, 200 nm, and 350 nm in healthy mice [23]. As shown in Figure 3A, a series of liposomes with the polyethylene glycol (PEG) of different chain lengths (1, 2, and 5 kDa) and concentrations (5, 10, and 20% *w/w* of total lipid) are prepared with different formulas to evaluate their pharmacokinetic properties. The evaluation results showed that longer circulation time could greatly enhance the passive tumor-targeted possibility of liposomes.

In addition, synthetic lipid molecules with tailored functionality for delivery applications, including ionizable cationic lipids and PEG lipids, have been prepared using various chemical techniques [24]. In aqueous solvents, lipid molecules can be self-assembled into various nanostructures to isolate their hydrophobic domain from the surrounding aqueous environment while also minimizing adverse interactions between individual molecules (electrostatic repulsion and spatially restricted crowding). The structural characteristics of the lipids (e.g., head group, alkyl chain length, and unsaturation) as well as the molar ratio of lipid components in the mixture have an obvious influence on the morphology of the fully assembled nanostructures and the tumor-targeted ability of the liposomal carriers.

Most liposomes are modified by different modification groups to extend their blood circulation time in vivo. In addition, the alteration in the orientation of the lipid linkage also contributes to prolonging the blood circulation duration in vivo. According to previous research, 2-((2,3-bis(oleoyloxy)propyl)-dimethylammonio)ethyl ethyl phosphate (DOCPe) liposomes cannot adhere to 2-((2,3-Bis(oleoyloxy)propyl)dimethylammonio)ethyl hydrogen Phosphate (DOCP) liposomes, cells, or proteins. Li et al. compared the blood circulation time of DOCPe liposomes and DOCP liposomes in vivo [25]. As depicted in Figure 3B, researchers injected near-infrared fluorophore-labeled liposomes into the tail vein of mice and tracked their blood concentrations by measuring plasma fluorescence at various time points. The results have revealed that the blood circulation time of DOCPe liposomes was longer than that of DOCP liposomes, which could be due to the great resistance of macrophages toward DOCPe uptake. This long blood circulation time increases the potential for DOCPe liposomes to accumulate in tumor tissue through the mediation of the EPR effect, thereby enhancing their passive tumor-targeted effect.

The stiffness and mechanical properties of lipid membranes have an obvious influence on cellular uptake, extracellular diffusion, and tumor-targeted properties of the liposomes [26,27]. The stiffness of lipid membrane can be regulated by altering the chemical properties of the lipid tail or by incorporating cholesterol. Membrane stiffness can be reinforced upon the increase in tail length or incorporating cholesterol into liquid-phase lipids [28,29]. Schroeder’s team investigated the effect of lipid composition on the uptake of Phosphatidylcholine (PC) liposomes via triple-negative 4T1 breast cancer cells [30]. Their flow cytometry results showed that cellular uptake of 4T1 breast cancer increased with an increase in acyl chain length in the following order: 1,2-dipalmitoyl-sn-glycero-3-phosphocholine (DPPC) (18:0) > DPPC (16:0) > 1,2-dimyristoyl-sn-glycero-3-phosphocholine (DMPC) (14:0). They further showed that DMPC (14:0) and 1,2-dilauroylsn-glycero-3-phosphocholine (DLPC) (12:0) liposomes could destabilize cell membrane, thus leading to a decrease in cell survival. In contrast, liposomes with longer acyl chains such as DPPC (18:0) and DPPC (16:0) could promote tumor cell proliferation on account of lipid integration into the tumor cell membrane. Strong sclerosis of DMPC (14:0) membrane by doping with cholesterol also enhanced cellular uptake. Tang et al. produced estrone (ES)-modified gemcitabine (GEM)-loaded PEGylated liposomes (ES-SSL-GEM) for enhancing delivery efficiency by identifying estrogen receptors overexpressed on lung cancer A549 cells. The findings showed that ES-SSL-GEM, which has good stability, can reduce drug leakage during blood circulation and enhance tumor-targeted effect [31].

### 2.2. Polymeric Nanoparticles

Polymers are macromolecules consisting of multiple repeating units called monomers. Homopolymers are formed from identical monomer units, and copolymers are composed of two or more different monomer units, typically in a defined order, configuration, and structure. Polymers are chemically flexible molecular platforms (e.g., their size and structure). In addition, their functions can be altered by regulating their physicochemical properties. Typically, PEG, as a well-known and widely used biocompatible synthetic polymer, can inhibit non-specific interactions by modifying the surface of the substrate. The molecular weight and surface density of PEG largely affect its adsorption toward proteins. PEG modification can also prolong the blood circulation time of the drug carrier being loaded. Intravenously injected PEGylated nanoparticles (e.g., PEGylated liposomes and micelles) have a long blood circulation [32,33]. In addition, these PEGylated nanoparticles can passively accumulate at the tumor site through the mediation of the EPR effect. Therefore, PEGylated nanoparticles are excellent drug carriers in drug delivery systems (DDS) for oncotherapy.

**Figure 4:**
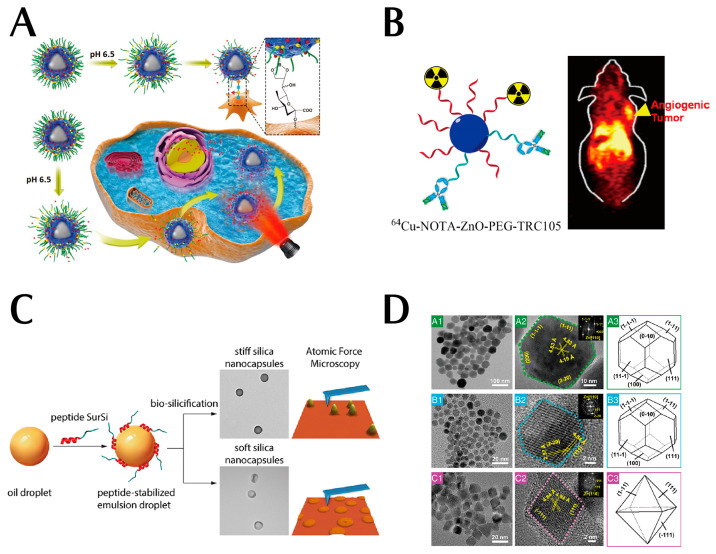
(**A**) A synergistic tumor-targeted, chemo-photothermal oncotherapy nanoplatform (PDA@CP-PEG) based on dynamic PEG. Reproduced with permission from Ref. [37]. Copyright 2018, John Wiley and Sons. (**B**) PET-imaging and red fluorescent ZnO nanoparticles by coupling with the tumor monoclonal antibody. Reproduced with permission from Ref. [43]. Copyright 2015, American Chemical Society. (**C**) The mechanical properties of nanoparticles. Reproduced with permission from Ref. [49]. Copyright 2018, American Chemical Society. (**D**) This combination of magnetic targeting, tumor penetration, and magnetic resonance imaging makes magnetosome-like MNPs very promising (A1 and A2 high-resolution electron micrographs and A3 three-dimensional morphology of natural magnetosome crystals from AMB-1. B1 and B2 high-resolution electron micrographs and B3 three-dimensional morphology of magnetosome-like MNPs. C1 and C2 high-resolution electron micrographs and C3 three-dimensional morphology of MNPs from the control reaction. The insets in A2, B2, and C2 show the corresponding fast Fourier transform patterns of the crystal structure). Reproduced with permission from Ref. [50].

Dendritic macromolecules as synthetic polymers have a controlled structure and molecular weight. Their surface density is easily controlled by dendrimers. The internal space and terminal functional groups of dendrimers facilitate the encapsulation and coupling of drugs or other therapeutic molecules. In addition to their effectiveness as drug carriers, dendrimers are also single-molecule nanoparticles and are smaller than other self-assembled organic nanoparticles (e.g., liposomes and micelles) [34]. Notably, PEGylated dendrimers can also prolong blood circulation [35,36], and their small size (8–20 nm in diameter) facilitates tissue penetration. The circulatory properties of dendrimers are largely influenced by the molecular weight of the modified PEG, the number of PEG molecules, and the generation of dendrimers. Fourth- and fifth-generation dendrimers fully modified with PEGylated polyamidoamine (PAMAM) of different molecular weights (2 kDa and 5 kDa) are retained in the bloodstream for a long time and accumulate to a lesser extent in normal organs. As shown in Figure 4A, Liu et al. reported a synergistic tumor-targeted chemo-photothermal oncotherapy nanoplatform (PDA@CP-PEG) based on dynamic PEG, borate ligand polymer-coated PDA nanoparticles. Due to the pH-responsive interaction between the PBA moiety and the catechol-containing PEG molecule, the resultant PDA@CP-PEG nanocarriers exhibit ‘passive targeting’ based on PEGylation and ‘active targeting’ by the exposed PBA moiety under weakly acidic conditions. The synergistic tumor-targeted effect of the exposed PBA moiety is demonstrated in both in vitro and in vivo studies. Both in vitro and in vivo studies jointly confirmed the relatively low systemic toxicity, effective tumor-targeted ability, and strong tumor-suppressive chemothermal activity of the multifunctional nanoparticles [37]. This is strong evidence of the excellent passive targeting ability of PEG polymers. Moreover, these multifunctional nanoparticles with synergistic tumor-targeted properties offer combined therapeutic strategies that will provide insights into the design of efficient antitumor nanoplatforms with potential clinical applications. Lin et al. conjugated porous polymers as a new class of polymeric materials with unique properties, presenting the synthesis of a new family of olefin-linked conjugated porous polymers through the Knoevenagel condensation of the key monomer 1,1′,6,6′-tetramethyl-[3,3′-bipyridine]-1,1′-diium iodide with different aryl aldehyde derivatives, such as cationic porous polymers that enable the inhibition of bacterial growth and exhibit good antibacterial properties against Gram-negative bacteria (e.g., *E. coli*) [38].

### 2.3. Metal Oxide Nanoparticles

Metal oxide nanoparticles have attracted much attention because of their unique physical properties. Among these, zinc oxide (ZnO) nanoparticles not only have a high specific surface area and good interaction with electromagnetic waves but also have tunable electrical properties [39]. ZnO nanoparticles have been developed in diverse forms including nanowires, nanotubes, and nanorods, which can be applied in different fields for specific purposes [40]. Their widespread applications span across optics, electronics, drug delivery, and biomedical imaging, which reflect their versatility and potential for various cutting-edge technologies [41]. It should be pointed out that one noteworthy feature of ZnO nanoparticles is the presence of hydroxyl groups (-OH) on their molecular surface, and they can slowly dissolve under both acidic (e.g., tumor microenvironment, TME) and strong alkaline conditions after direct contact with aqueous solutions. Moreover, zinc is an essential trace element. Therefore, zinc-based nanoparticles have excellent biosafety [42].

As shown in Figure 4B, Hong et al. developed tumor-targeted PET imaging and red fluorescent ZnO nanoparticles by coupling them with the tumor monoclonal antibody TRC105 as a potential thermosensitive with wide application potential. Notably, scanning electron microscopy images showed the size range of nanoparticles. In addition, to improve the stability of ZnO nanoparticles in the biological environment and reduce ionization, authors modified the PEG on the surface of the ZnO nanoparticles. Moreover, such modification is beneficial for improving the blood circulation time of ZnO nanoparticles. Scanning electron microscopy, differential scanning calorimetry, and zeta potential jointly confirmed the successful surface functionalization [43]. Moreover, these nanoparticles have suitable nanoscale dimensions, excellent stability, and good homogeneity, which greatly enhanced their passive tumor-targeted ability. Besides, the introduction of TRC105 endowed ZnO nanoparticles with an active tumor-targeted property, which further enhanced their tumor accumulation.

### 2.4. Silicon Dioxide Nanoparticles

As is well known, the engineering of the physicochemical properties of nanoparticles is important for the successful development of targeted drug delivery systems. Additionally, the size, shape, charge, interactions, chemical composition, and surface properties of nanocarriers play an important role in immune evasion, blood circulation, tumor infiltration, penetration, etc. [44]. As shown in Figure 4C, the mechanical properties of nanoparticles (often expressed as hardness or Young’s modulus) are one of the other physical properties that have attracted attention due to their role at the nano-biological interface [45,46,47,48]. Understanding the mechanical properties of nanoparticles and their effects on passive and/or active targeting is necessary for the rational design of optimal drug delivery nanocarriers.

In view of this, Hui et al. have synthesized silica nano-capsules with different hardness and modified them with PEG and FA-PEG to investigate the impact of the mechanical properties of the nanoparticles on their biological properties, including nonspecific and ligand-receptor-mediated cellular uptake and passive/active tumor targeting in vivo. In this study, it was found that macrophages increased the uptake of hard nano-capsules through a non-specific pathway, and the hard nano-capsules were internalized by tumor cells with the assistance of the receptor. Also, the hardness of the nano-capsules may have an influence in their ability to cross the biological barrier. When subjected to 3D tumor spheroids, the nano-capsules tended to accumulate in marginal areas. In xenograft tumor models, the soft nano-capsules had a higher tumor aggregation efficiency than hard nano-capsules. In contrast, the abnormally high spleen clearance has been a major barrier toward hard nano-capsules with tumor targeting [49]. The experimental results demonstrate that nanoparticles with lower stiffness are less prone to be taken up by phagocytes during blood circulation and that their greater tissue penetration can greatly enhance the passive tumor-targeted ability of nanoparticles.

### 2.5. Magnetic Nanoparticles (MNPs)

Inspired by the biomineralization process observed in magneto-tropic bacteria, Ma et al. developed a novel approach by incorporating the amphiphilic Mms6 protein into a self-assembly anti-micellar beam system. The integration process led to the development of magnetic vesicle-like nanoreactors. The morphology, magnetic properties, and MR relaxation properties of the resulting MNPs were also characterized (see Figure 4D). Both the small size and a strong magnetic-targeted ability result in a significant enhancement of their penetration in tumor tissues by an order of magnitude. This enhancement has the potential to greatly improve their passive targeting efficiency. In addition, to endow the natural magnetic vesicle MNPs with good monodisperses, good water solubility, and small hydrodynamic size, nanoreactors are also encapsulated by DSPE-mPEG. Therefore, the integration of magnetic targeting, tumor penetration, and magnetic resonance imaging (MRI) renders magnetosome-like MNPs very promising for potential nanomedical applications [50]. Typically, ferroptosis is a programmed cell death process that is dependent on iron. This process is characterized by the accumulation of intracellular reactive oxygen species (ROS) [51,52,53]. In this process, Fe^2+/3+^ ions could react with hydrogen peroxide (H_2_O_2_) to generate ROS via the Fenton reaction. ROS can induce lipid peroxidation of polyunsaturated fatty acids to generate lipid peroxides (LPO), which could destroy the integrity and structure of cells. In addition, iron oxide nanoparticles are one of the popular magnetic-targeted delivery carriers in recent years. Iron oxide nanoparticles not only enhances passive tumor targeting with their magnetic targeted delivery capability but also enhances the tumor-killing effect of nanomedicines via the ferroptosis mechanism, which is a ‘win-win delivery carrier’. Zhou et al. prepared T1-weighted MRI point-like core–shell Fe_3_O_4_/Gd_2_O_3_ heterogeneous nanoparticle (FGNP) contrast agents based on very small magnetic iron oxide nanoparticles (ES-MIONs), loaded with sorafenib (SFN) and self-assembled into SA-SFN-FGNP by embedding mPEG-PPS-NH_2_ on the surface of FGNP. After SA-SFN-FGNP was internalized into tumor cells, acidic TME. In addition, a burst release of SFN and Fe^2+/3+^ from SA-SFN-FGNP can be initiated under endosomal conditions. The released SFN increased H_2_O_2_ levels by inhibiting systemic XC and decreasing GSH levels. Both H_2_O_2_ and Fe^2+/3+^ accelerated the Fenton reaction. Disassembly can further significantly accelerate the rapid release of SFN and Fe^2+/3+^. The aforementioned cyclic reactions resulted in an intensified cycle of ROS generation for enhancing tumor ferroptosis [54]. The results show that magnetic targeted delivery can also improve the efficiency of nanomedicines.

## 3. Active Targeting of Drug Delivery

**Figure 5:**
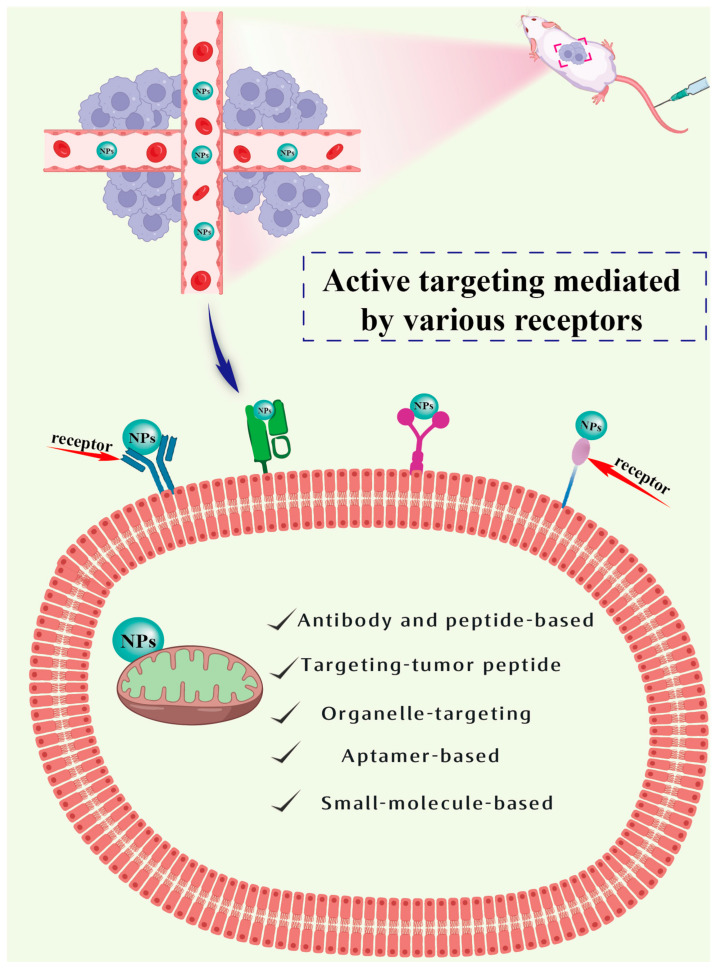
Mechanism of active targeting and types of modifying ligands.

Active targeting involves the alteration of particular ligands, antibodies, or other molecules on the surface of nanoparticles to identify and attach to specific cells or tissues at the targeted site, thereby providing more accurate drug delivery. For instance, the utilization of nanoparticles adorned with antibodies can identify and combine with specific antigens present on the exterior of tumor cells, thus enabling more precise drug delivery. Active targeting is mainly divided into antibody-based targeting, peptide-based targeting, aptamer-based targeting, and small-molecule-based targeting (see Figure 5). The active targeting of nanoparticles in vivo first follows the EPR effect and then, through a bypass pathway, reaches the specific tumor site with surface modifications.

### 3.1. Antibody-Based Targeted Nanoparticles

In the pursuit of finding suitable ligands, antibodies were selected as the most popular ligand for the advancement of targeted ligand development [55]. Antibodies present on the surface of the nanoparticles can be specifically recognized and bound to antigens on the surface of tumor cells, thereby achieving the active targeting delivery of nanoparticles. Most antibodies have the ability to induce cell death via the primary direct mechanism by inhibiting growth factor receptor signals. The mechanism of monoclonal antibody (mAb) action involves components of the host immune system, including complement-dependent cytotoxic effects (CDC), antibody-dependent cytophagy (ADCP), and antibody-dependent cell-mediated cytotoxic effects (ADCC). To make better use of antibodies for targeted therapy, the most ideal antigen needs to be screened, and it should be specifically overexpressed on tumor cells, not on normal cells. Based on the rapid development of antibodies and further research into their mechanisms of action, different conventional antibodies such as anti-epidermal growth factor receptor (EGFR), anti-vascular endothelial growth factor (VEGF), and anti-programmed death ligand 1 (PD-L1) antibodies, have enabled various therapeutic applications. A variety of antibodies as well as antibody-drug couples (ADC) have been registered and approved for targeted drug delivery such as rituximab for non-Hodgkin’s lymphoma [51], trastuzumab for breast cancer [52], bevacizumab for angiogenesis inhibition [53], and cetuximab for advanced colorectal cancer [54]. Generally, monoclonal antibodies in immunotherapy are used in two primary approaches. Firstly, it targets the antigen associated with the surface of the tumor cells and the antigen–antibody combination allows the antibody-carrying adjuvants to be delivered to the tumor cells for precise therapeutic effect. Secondly, it targets the co-stimulatory molecules on the surface of the immune cells, leading to direct immune stimulation to enhance the immune response at the tumor site.

Some antigens on the tumor cell surface are specific molecular structures existing on the surface of tumor cells and can be recognized by the immune system as foreign bodies to provoke an immune response. These antigens may be caused by mutations or abnormal expression of tumor cells. Tumor cell surface antigens can be divided into two distinct categories: tumor-associated antigens (TAA) and tumor-specific antigens (TSA). TAA is expressed in both normal tissues as well as at high levels in tumor cells while they induce a tumor patient-specific T cell immune response in tumor patients [56,57], as a potential targeted structure for specific immunotherapy. Seeger et al. discovered that glycosides deaminate (GD2) are tumor-associated antigens that are highly expressed in all neuroblastoma cells and enhanced anti-tumor immunotherapy with immunotherapy using chimeric anti-GD2 monoclonal antibodies in combination with IL-2 and GM-CSF. TSA is only expressed in tumor cells and has little or no expression in normal tissues [58]. More immunogenic and less tumor toxic than TAA, TSA is often associated with mutations or aberrant expression in tumor cells. Detection of TSA can be used in personalized therapy and immunotherapy approaches. Yang et al. identified a highly specific tumor cell surface antigen in mesothelioma. A phage antibody display library was used to identify a specific antibody M25 that binds to all subtypes of mesothelioma, and its targeted antigen is human alkaline phosphatase placenta-like 2 (ALPPL2) [59]. The targeted antigen ALPPL2 is highly tissue-specific and is expressed only in mesothelioma. The microtubule inhibitor is coupled to anti-ALPPL2 human mAbM25 to create an ADC, and the M25 ADC has been shown to successfully inhibit tumor cell proliferation in vitro and the growth of mesothelioma cell line allografts in vivo.

The specificity and diversity of tumor cell surface antigens form the foundation for individualized therapy, and the identification and utilization of these antigens allow for the development of immunotherapies, targeted therapies, and vaccines for specific antigens. At present, the study of tumor cell surface antigens is still in progress to better understanding tumor immunology and to improve the effectiveness of oncotherapy.

The stimulation of T cells and B cells requires a dual signaling pathway, with the co-stimulatory molecule being the second signal that allows T cells and B cells to proliferate [60]. The absence of the second signal (the costimulatory molecule) results in an incompetent state of T cells [61]. TME is an immunosuppressive environment where T cells are in an incompetent state due to the lack of costimulatory molecules. Li et al. demonstrated that CD137, a member of the tumor necrosis factor (TNF) receptor superfamily, is an activation-inducible T cell costimulatory molecule with strong anticancer efficacy in combination with other anticancer drugs in non-immunogenic or poorly immunogenic tumors. The monoclonal antibody, which targets CD137, has shown significant therapeutic efficacy in the treatment of melanoma using monoclonal antibodies [62].

### 3.2. Peptide-Based Targeted Nanoparticles

Peptides are formed through the condensation of amino acids through amide bonds. The diversity of amino acids and the multiple arrangements of amino acid sequences give rise to a large number of peptides with a wide range of biological functions. Most peptides are part of protein structure that are hydrolyzed from proteins into peptides and still maintain the properties of proteins [63]. These give peptides good biocompatibility and potential for biodegradability, as well as chemical modifiability. Meanwhile, peptides are small in size, have low immunogenicity, low cost, and are easy to manufacture. On account of peptides with the many advantages mentioned above, they have become very attractive targeted molecules. Ideally, peptides should specifically target proteins that are overexpressed in tumor cells. Peptides capable of hosting specific proteins have been explored in multiple different sequences using phage display techniques for tumor-targeted systems. The most commonly used phage display technique for screening peptides in various settings (in vivo, in vitro, in situ, and ex vivo) [64] involves the following steps: first, phage libraries are introduced into established model mice through systemic tail vein injection, where they are allowed to bind in vivo for a specific period. Subsequently, unbound phages are washed out through perfusion, and the phages preferentially bound to targeted tissues are collected, partially homogenized, and centrifuged to obtain phages for subsequent rounds of biological screening. Following perfusion and washing to remove unbound phages, the targeted tissues with preferentially binding phages were collected from the mice. The tissues were then partially homogenized and subjected to centrifugation to obtain phages for subsequent rounds of bio-screening. Following 3–5 rounds of biological screening, the number of specific phages increases, and the overexpression of a peptide at a specific site is demonstrated through methods such as high-throughput genome sequencing, which guides subsequent experiments [65,66]. In vitro screening involves the direct co-cultivation of the phage library with the same cell line, followed by incubation for a specific period. Subsequently, unbound phages are washed away, and the cells are lysed to obtain intracellular phages. These phages are then amplified for use in subsequent rounds of screening [67]. The phage display technology for screening specific peptides can be divided into the following general steps: the first step, construction of a phage library for peptide display; the second step, incubation of the phage library with the targeted site for a specific period to allow binding; the third step, removal of unbound and non-targeted specific phages through repeated washes; the fourth step, elution of the phages; the fifth step, amplification of specific phages for use in the next round of bio-screening [68]. Scodeller et al. conducted in vivo peptide phage display in mice with 4T1 metastatic mammary tumors to identify peptides that target peritoneal macrophages. Model mice were injected with a phage library expressing 9-amino acid cyclic CX7C peptides. Two hours later, peritoneal cells were collected to obtain bound phages by amplification. The resulting phages were then subjected to high-throughput sequencing of peptide-encoding fragments of the phage genome, ultimately leading to the identification of the short peptide CSPGAKVRC, which was selected for use in the therapy of breast cancer in the mammary carcinoma model [69]. There are two main strategies for modifying nanoparticles with peptides. Firstly, the targeted peptide is modified on the surface of the nanoparticles, e.g., the antitumor peptide arginine–glycine–aspartate (RGD), transferrin receptor binding peptide (TfR-BP), etc. Secondly, the targeted peptide is carried inside the nanoparticles. For example, the classical pH-responsive PEG-poly (L-aspartate) block copolymer. Peptide-targeted sites in vivo include three different types: firstly, tumor-targeted peptides (various tumor cell surface receptor targeting), secondly, TME-targeted peptides (including tumor vascular system targets, tumor extracellular matrix (ECM) targets, and tumor-associated cell targets), and thirdly, subcellular organelle-targeted peptides (plasma membrane, nucleus, mitochondria, etc.) [70].

**Figure 6:**
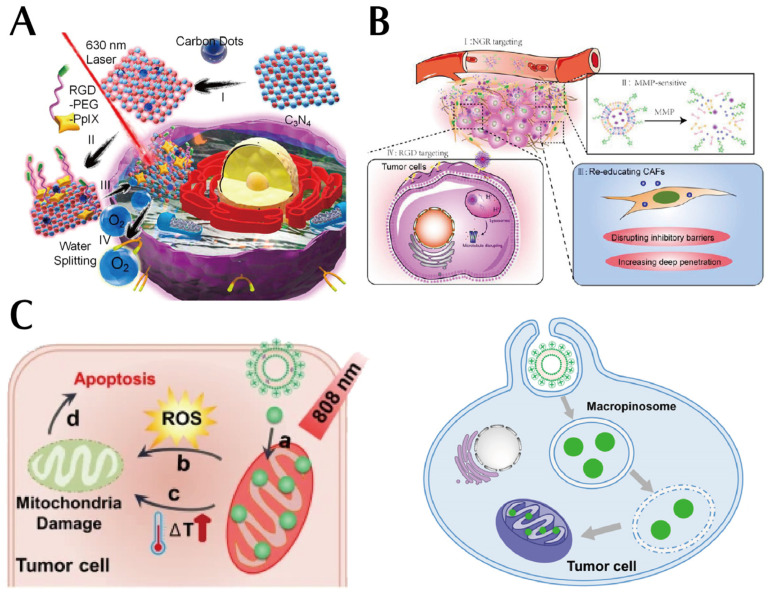
(**A**) Integrin’s extracellular region of the *α* chain can specifically recognize the RGD sequence-containing peptide and mediate the adhesion of integrin to the extracellular matrix. Reproduced with permission from Ref. [78]. Copyright 2016, American Chemical Society. (**B**) A dual-targeted hybrid micelle–liposome system (RPM@NLQ), resulting in sequentially delivered Que and PTX for fibrotic TME remodeling and chemotherapy enhancement. Reproduced with permission from Ref. [84]. Copyright 2022, American Chemical Society. (**C**) A delivery platform for targeted delivery to mitochondria to enhance tumor accumulation and cell permeation(a is mitochondrial targeting, b is ROS production, c is PTT-induced hyperthermia, d is apoptosis induced by mitochondria-targeted treatment.). Reproduced with permission from Ref. [97].

Tumor-cell-targeted peptides can bind to specific proteins (cell surface receptors, growth factor receptors, and cell adhesion molecules) on the tumor cell membrane or secreted from cells that promote tumor proliferation, invasion, migration, and migration, thus allowing drugs loaded on nanoparticles to have an anti-tumor effect. EGFR is an example of a protein that is expressed in normal human cells but is overexpressed in epithelial tumors [71]. EGFR plays an important role in the proliferation, differentiation, and migration of tumor cells. Anti-EGFR mAb induces EGFR internalization in colorectal cancer (CRC) cells. TGF-*α* is a ligand for EGFR, and TGF-*α* induces EGFR internalization in CRC cells. Two monoclonal antibodies targeting EGFR, cetuximab and panitumumab, have been shown to be effective in combination with chemotherapy or as monotherapy, with cetuximab-sensitive CRC cells displaying enhanced EGFR internalization, stronger cell growth inhibition, and more enhanced apoptotic signaling [72,73]. Integrins are transmembrane heterodimeric glycoproteins formed by non-covalent bonds between two peptide chains, *α* and *β*. The extracellular region of the *α* chain specifically recognizes peptides containing RGD sequences [74]. Integrin *α*v*β*6 is a member of the integrin family, and it is particularly expressed on the surface of numerous tumor cells, is not present in normal tissues, and plays a crucial role in the proliferation [75], adhesion, migration, and invasion of tumor cells. In view of this, integrin *α*v*β*6 serves as an ideal specific target. Li et al. showed that a peptide called H2009.1 was identified in the non-small cell lung cancer (NSCLC) cell line H2009 and from the phage display technology peptide library. The 2′-position of paclitaxel is utilized to form an ester bond with the tetrameric peptide, leading to the production of a paclitaxel-H2009.1 peptide coupling, which selectively transports the chemotherapeutic agent paclitaxel to *α*v*β*6 positive cells, resulting in antitumor activity that is comparable to that of paclitaxel alone [76]. In Figure 6A, integrin *α*v*β*6 is absent in normal pancreatic cells, however, it is present in pancreatic ductal adenocarcinoma (PDAC) cells, making it an ideal target for the treatment of this tumor. Elizabeth et al. used the DNA-binding compound tesirine coupled to the *α*v*β*6-targeted peptide A20FMDV2 to demonstrate antitumor efficacy against *α*v*β*6-positive human PDAC allografts in an immunodeficient mouse model [77]. Li et al. synthesized a multifunctional molecule consisting of an integrin-targeted peptide, a cytotoxic platinum (IV) prodrug, and a fluorescent or affinity probe linked to a flexible linker using a combination of solid-phase peptide synthesis and chemo selective linkage, a peptide–drug multiplex was designed to enhance the specificity of oncotherapy and imaging, as well as to achieve higher drug loading [78].

TME has many different physicochemical properties compared to the normal human internal environment, especially its low oxygen, low pH, and high-stress characteristics [79], it includes all non-malignant host cells and non-cellular components [80,81], and it includes, without limitation, the immune system, blood cells, endothelial cells, adipocytes, and stroma [82]. Also, TME has a large number of growth factors, cell chemokines, and diverse protein hydrolases in TME, which are extremely favorable for tumor proliferation, invasion, adhesion, and angiogenesis. TME in PDAC is highly immunosuppressive and pro-connective tissue proliferative, and one of the crucial molecules leading to immunosuppression and fibrosis is TGF-*β*. Maria et al. tracked immunosuppression and fibrosis in PDAC with a unique immunomodulatory vaccine containing a TGF-*β*-derived peptide TME. The vaccine with the TGF-*β* derived peptide increased infiltration of CD8^+^T cells and the ratio of intra-tumoral M1/M2 macrophages and decreased the expression of myofibroblast-like cancer-associated fibroblasts (CAF) related genes and expression of genes encoding fibroblast-derived collagen [83]. The vaccine ultimately shown to target immunosuppression and fibrosis in TME by polarizing the cellular components toward a more hyperinflammatory phenotype. CAF is an important stromal cell in TME and functions to create a physical barrier for drug delivery and permeation of tumor tissue. Therefore, effectively down-regulating CAF to disrupt the physical barrier may enhance the penetration and accumulation of therapeutic drugs, thereby improving treatment outcomes. As depicted in Figure 6B, Duan et al. co-encapsulated micelles of antifibrotic quercetin (Que) and small-sized RGD modified paclitaxel (PTX)-loaded (RPM) into enzyme-sensitive stimulus-responsive liposomes and further modified the liposomes with NGR peptide (NL) as targeted groups. A dual-targeted hybrid micelle–liposome system (RPM@NLQ) results in sequentially delivered Que and PTX for fibrotic TME remodeling and chemotherapy enhancement. Subcellular organelle-targeted cancer therapy (STCT) is one of the latest developments in tumor nanomedicines [84], targeted therapeutics to specific organelles for treatment, allowing higher concentrations of therapeutics to accumulate in specific subcellular compartments (e.g., cell membrane, mitochondria, and nucleus). It plays a critical role in maintaining cell membrane integrity, cellular internalization, and protection of living cells; if the integrity of the cell membrane is disrupted this can lead to reduced permeability and consequently increased accumulation of drugs that can be useful in oncotherapy.

In comparison with traditional tumor treatment strategies, tumor treatment strategies that target the cell membrane are not affected in terms of drug accumulation due to the effectiveness of cellular endocytosis [83]. The nucleus is the control center of the cell and acts as a storage reservoir for genetic information and has a critical role in cell proliferation [85]. The nucleus is isolated from the rest of the cytoplasm by the double-membrane structure of the nuclear envelope (NE), which is a structure that constitutes the main barrier and major rate-limiting step for drug transport to the nucleus. The nuclear pore complex (NPC) on NE is a bridge for material exchange [86]. Small molecules can freely enter and exit the NPC, while large molecules with molecular weights > 50 kDa or diameters > 9 nm cannot pass through [87]. The specific nuclear targeting of the NPC not only requires their small size of the nanoparticles but also the specific and efficient nuclear binding ability of the nanoparticles [88]. Sahin et al. constructed DOX-loaded poly (3–hydroxybutyrate–co–3–hydroxy valeric acid) nanoparticles and attached 17 a.a. peptide nuclear targeting molecules (NLS) to the nanoparticles loaded with adriamycin to target the nucleus, although nanoparticles > 50 nm in size did not translocate into the nucleus, their attachment to the nuclear membrane concentrated the anticancer agent around the nucleus and increased its effect, resulting in a significant upregulation of the number of tumor cells due to apoptosis [89]. Mitochondria are a source of cellular energy. The presence of various metabolic intermediates and energy in mitochondria provides tumor cells with the necessary metabolic raw materials for their rapid proliferation [90]. Mitochondria play a regulatory role in the apoptosis of cells [91,92]. Altered mitochondrial function is a hallmark of many tumors [93,94], and Warburg found that tumor cells are more dependent on glycolysis to produce ATP [95], even when the cellular environment is rich in oxygen. The Warburg effect represents a remarkable metabolic feature of tumor cells. Mitochondria are reduced in many tumors, and mitochondrial respiratory dysfunction due to mutations/deletions in mitochondrial DNA (mtDNA) plays a significant role in the metastasis of tumor cells [96]. As illustrated in Figure 6C, Jiang et al. assembled mitochondrial-targeted liposomes (L-G2R-DA) with dendritic lipopeptides, soy phosphatidylcholine (SPC), cholesterol, DSPE-PEG, and the photosensitizer indocyanine green (ICG) co-assembled into a delivery platform for targeted delivery to mitochondria to enhance tumor accumulation and cell permeation [97].

### 3.3. Aptamer-Based Targeted Nanoparticles

Nucleic acid aptamers are short, single-stranded RNA or DNA oligonucleotides [98]. Nucleic acid aptamers can be used for a diversity of targets (e.g., proteins, phospholipids, small molecules, sugars, and viruses), which are selected using a live cell-based exponential enrichment ligand phylogenetic technique (Cell-SELEX) for oligonucleotide sequences that associate with multiple types of targets with high affinity and specificity through various types of interactions (e.g., van der Waals forces, hydrogen bonds, salt bridges, hydrophobic, polar groups, stacking interactions, and other electrostatic interactions) [99]. Compared to antibodies, aptamers have many advantages, (1) aptamer screening is efficient, taking only a few days to a few months; (2) corresponding antibodies cannot be found for toxins or less immunogenic antigens, but aptamers can be considered [100]; (3) solid-phase synthesis of nucleic acids is more mature, less expensive, and with minimal batch variation; (4) aptamers are easier to modify; (5) aptamers are more thermally and chemically stable [101]; (6) smaller molecular weight and improved tissue permeability; and (7) aptamers are rarely immunogenic and do not cause immunological side effects [102]. There are three types of nucleic acid aptamers, those that target tumor cell surface proteins, the complement system, and the immune system.

**Figure 7:**
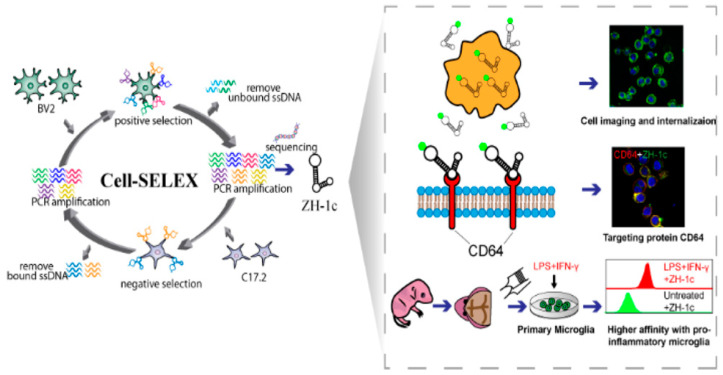
Used the Cell-SELEX technology to develop a DNA adaptor ZH-1. Reproduced with permission from Ref. [104]. Copyright 2023, American Chemical Society.

Cell surface proteins are a major pathway of communication between cells and their external environment. Membrane proteins are the main sensors of extracellular stimuli and initiators of intracellular signaling, and their abundance on the cell surface is often dynamically regulated [103]. Cell surface proteins make them one of the most desirable targets at the center of targeted therapies. As shown in Figure 7, Zhu et al. used the Cell-SELEX technology to create a DNA adaptor ZH-1 that binds microglia with high affinity and is internalized by microglia with good serum stability after modification. Meanwhile, the binding target of ZH-1c on microglia is the transmembrane protein CD64, which is increased in response to inflammatory stimulation by lipopolysaccharide and interferon-*γ*, thus enhancing the affinity of ZH-1c for activated microglia and serving as a novel and effective molecular tool for diagnostic and microglia-targeted therapy [104]. Sarah et al. use SELEX technology to identify RNA aptamers that specifically associate with the tumor stem cell marker CD133 (a highly glycosylated membrane glycoprotein), which is effectively internalized by CD133-positive tumor cells and displays superior invasion of tumors [105].

The complement system is an invaluable component of the human immune system and plays a critical role in innate immunity [106]. It can be activated through three pathways, the classical pathway, the alternative pathway, and the lectin pathway [106]. These three pathways all eventually fall into one pathway, and complement component 5 (C5) is activated as C5a and C5b, which binds to C6 and C7 to form the C5b-C6-C7 compound. This compound anchors to the cell membrane and interacts with C8 and C9 to form the membrane attack complex (MAC), which in turn disrupts the cell membrane to promote phagocytosis to recognize and destroy the ‘invader’ [107]. In addition, the complement system is able to maintain immune balance in the body by regulating the immune response [108]. Throughout the progression of the tumor, complement regulatory protein (CRP) is overexpressed to resist attacks by activated complement [109]. C5 is activated into C5a and C5b, and C5a is a systemic allergenic toxin that is essential for the general inflammatory response by inducing the migration and activation of immune cells toward and at the site of infection. The C5a is considered to be the most potent chemoattractant in the body [110]. John R et al. observed with fluorescence confocal microscopy that the biotinylated DNA aptamer of MUC1-5TR-1 can modify the plasma membrane of human breast cancer (MCF7) cells, initiating the classical complement pathway, leading to the anchoring of complement on target cells through streptavidin–C1q complexes that synergistically target different surface-bound tumor markers of the aptamer–C1q complex mixture, which can inhibit CRP on MCF7 cells and provide antitumor efficacy [111].

The immune system is a defense mechanism that protects the body against foreign pathogens and protects it against infection and disease. It consists of a complex system of cells, molecules, and tissues that work together to coordinate against invasion by bacteria, parasites, viruses, fungi, etc. [112]. The immune system is divided into two main components: innate and acquired immunity [113]. Innate immunity is the non-specific intrinsic immunity that includes dendritic cells (DC), macrophages, granulocytes, mast cells, natural killer (NK) cells, etc. It is the first line of defense against pathogens and foreign substances and quickly detects danger signals through the expression of the pattern recognition receptor (PRR) family [114,115]. Acquired immune responses are specific immune responses that are generated by exposure to a specific pathogen and have a memory of the specific pathogen to rapidly provoke an efficient immune response upon pre-exposure. The acquired immune response involves T-lymphocytes, B-lymphocytes, etc. [116]. Hu et al. discovered that activated cell phosphatase 1 (PAC1) functions as an immune checkpoint to down-regulate the anti-tumor activity of T cells, screened an aptamer for PA5 with high affinity and specificity for PAC1 using the SELEX system, and developed a dual module aptamer, PAC1-AS, consisting of a cellular internalization module and a targeted module to explore and develop effective oncotherapy [117]. The pancreatic ductal adenocarcinoma (PDAC) constitutively expresses the G protein-coupled cholecystokinin B receptor (CCKBR) and dual SELEX selection against ‘exposed’ CCKBR peptides, and CCKBR-expressing PDAC cells screens for the biofouling of AP1153 to the surface of fluorescent particles, greatly facilitating the delivery of nanoparticles to PDAC cells in vivo [118]. Chen et al. showed that in triple-negative breast cancer (TNBC), the ssDNA aptamer PDGC21-T as a probe to target the ligand and NK cells were used as a therapeutic agent to generate aptamer-engineered NK cells to reduce the number of TNBC cells through apoptosis [119].

### 3.4. Small-Molecule-Based Targeted Nanoparticles

**Figure 8:**
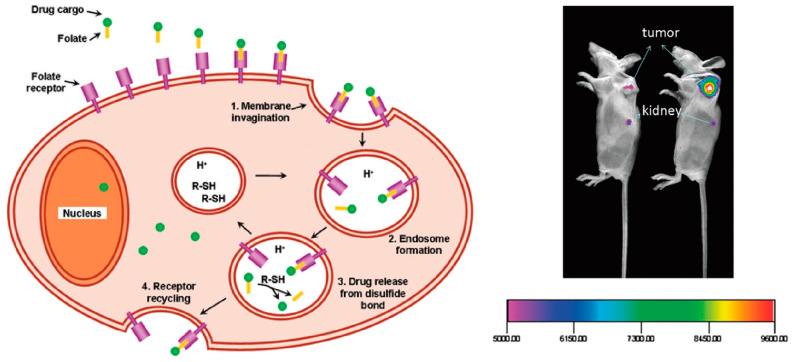
Folic acid has a high affinity for the folate receptor and does not dissociate from the receptor although it enters the cell. Reproduced with permission from Ref. [121]. Copyright 2010, American Chemical Society.

Small-molecule-targeted nanoparticles are actively targeting delivery systems that combine small-molecule compounds with nanoparticles to achieve a strategy for precise targeted delivery [120]. The specific properties and surface modifications of nanoparticles are used to effectively deliver drugs to specific cells or tissues, thereby enhancing therapeutic efficacy and minimizing side effects. A significant advantage of small molecule nanoparticles is their small size, which enables them to better cross biological barriers in the body and facilitate efficient drug delivery. Folic acid is a member of the B vitamin family, whose receptors are overexpressed on tumor cells, and folic acid can be used as a ligand to bind with high affinity to the receptor on the tumor for targeted delivery to the tumor site (see Figure 8) [121]. Joanna et al. used *β*-cyclodextrin-modified quantum dots as a carrier and folic acid as a targeted ligand for drug delivery to tumor tissue, synthesizing the nanocomplex QDs-*β*-CD-FA; the addition of FA significantly enhanced the endocytosis of nanoparticles by tumor cells [122].

## 4. Comparison and Combination of Passive and Active Targeting

### 4.1. Comparison of Passive and Active Targeting

The two modes of targeted nanoparticles in vivo, active and passive targeting, have their advantages and disadvantages in oncotherapy. The EPR effect plays a primary role in passive targeting, as being in a state of hypoxia in the tumor microenvironment increases the infiltration of blood vessels within the tumor [123]. The absence of lymph node drainage inside the tumor hinders the delivery of nanoparticles into the lesion, and the presence of the EPR effect enables nanoparticles to accumulate and concentrate inside the tumor, facilitating their anti-tumor effect [20]. Subsequently, due to the limitations of passive targeting, precision active targeting was developed with the introduction of precision medicine. Active targeting also has an EPR effect, but most importantly, the surface of the nanoparticles is modified with antibodies, peptides, aptamers, etc. This modification enables precise targeting based on extended pharmacokinetic times and significantly enhances therapeutic efficacy.

The first advantage of passive targeting is its universality, as it relies on the physical properties of tumor tissue to achieve drug enrichment, making it applicable to a wide range of tumor types. The second advantage is its improved bioavailability, as coupling the nanocarriers to the drug extends the metabolism time in vivo and avoids faster clearance. The third advantage is selective accumulation, as the main effect of passive targeting is the EPR effect, which relies on the physical difference between the tumor site and normal tissue to achieve the selective accumulation of drugs and reduce the adverse effects of drugs on normal tissue. The fourth advantage is enhanced local efficacy, as the nanocarriers are loaded with drugs and concentrated at the tumor site, significantly increasing the concentration of single drugs at this site and enhancing local drug efficacy at that specific location. The fifth advantage is the reduction in toxic side effects. Passive targeting allows for better drug accumulation at the tumor site, thereby minimizing toxicity to normal tissues. The sixth advantage is the simplicity of manufacturing, making it a convenient approach for drug delivery. Passive targeting also has a few limitations. The degree of targeted is limited, it relies on physiological differences between tumor tissue and normal tissue, and these differences may be influenced by various drugs or vectors, which may lead to reduced targeted efficiency, it is affected by tumor heterogeneity, and the heterogeneity of tumor tissue may lead to uneven enrichment of the drug in different areas [124], thus affecting the delivery effect, and passive targeting may not be suitable for all tumor types.

Based on the limitations of passive targeting, active targeting has become increasingly popular among researchers, and there has been an increasing number of studies on active targeting in recent years, with a growing variety of active targeting nanocarriers. Active targeting offers numerous advantages, one of them being high specificity. Through the specific binding of ligands to corresponding receptors, active targeting achieves precise and highly specific drug delivery, significantly enhancing targeted efficiency. Two is enhanced delivery efficiency—the precise delivery of active targeting increases the drug delivery efficiency in the tumor tissue, improving the therapeutic effect. Three is controlled—active targeting nanoparticles can be adjusted, and active targeting nanoparticles can be custom designed by adjusting the properties of ligands and carriers to meet different therapeutic needs. However, there are also some disadvantages, in that the selection and modification of targeted molecules become more complex, increase the complexity and cost of preparation, a certain off-targeted rate, and the heterogeneity of tumor sites may affect the effectiveness of targeted delivery.

In summary, passive and active targeting have their advantages and disadvantages, and if combined with passive and active targeting delivery strategies, they can be synergized to achieve more efficient delivery.

### 4.2. Combination of Passive and Active Targeting

Active and passive targeting can work together synergistically to enhance the effectiveness and selectivity of drug delivery. Their synergistic interaction can be achieved in the following ways—enhanced targeted active and passive targeting can be applied simultaneously in DDS to enhance drug accumulation in target tissues or cells. Active targeting is accomplished through the introduction of specific ligands or antibodies that can directly bind to receptors on the surface of the target cell, making for more precise targeting delivery. Passive targeting uses the properties of the disease area or abnormal tissue to achieve selective accumulation. By combining active and passive targeting strategies, the efficiency and selectivity of drug delivery to target tissues or cells can be significantly improved, leading to enhanced therapeutic outcomes. Active targeting can selectively deliver drugs to specific cell subpopulations or subcellular organelles to achieve more precise therapeutic effects. On the other hand, passive targeting allows for the selective accumulation of multiple types of cells or tissues by modifying the physical and chemical properties of the nanoparticles. In this way, the combination of active and passive targeting can expand the range of drug delivery while meeting the requirements of various levels and types of therapy. The drug delivery process often encounters biological barriers, including clearance by the immune system, drug metabolism, and elimination. The synergistic effect of active and passive targeting can effectively address these challenges and overcome delivery barriers. Active targeting helps the drug evade clearance and metabolism by the immune system, increasing the stability and durability of drug delivery. By introducing specific targeted ligands or antibodies, active targeting enables the drug to selectively bind to receptors on the target cells, avoiding premature clearance. Passive targeting enhances drug accumulation in disease areas and reduces drug distribution in non-target tissues, thus enhancing delivery. Overall, active and passive targeting can work synergistically to improve the effectiveness and selectivity of drug delivery by enhancing targeting, expanding the range of delivery, and overcoming delivery barriers. This new synergy has great potential in the field of drug delivery. The combined action of active and passive targeting is significant in drug delivery. This synergistic strategy can overcome the limitations of a single approach and improve the effectiveness and selectivity of drug delivery. Active targeting is by binding targeted molecules to receptors on the surface of tumor cells to achieve specific recognition and delivery of drugs. This strategy allows the drug to be directly delivered to the tumor cells, increasing the local concentration of the drug and reducing the impact on normal tissue. However, there are some limitations to active targeting such as tumor heterogeneity and variability in receptor expression, which can lead to reduced delivery efficiency. Passive targeting makes use of the characteristics of tumor tissue to achieve the specific accumulation of drugs. Tumor tissue is often characterized by increased vascularity and permeability, which allows drug carriers, such as nanoparticles to accumulate in the tumor region. This strategy does not depend on specific receptor recognition and therefore has broader applicability. However, passive targeting also has its challenges. The tumor microenvironment, including the tumor mesenchyme, can impede drug penetration, limiting the overall effectiveness of drug delivery. Additionally, there may be inhomogeneity in drug release within the tumor, leading to uneven drug distribution and potentially diminishing therapeutic efficacy.

By combining active and passive targeting strategies, the limitations of each individual approach lead to improved drug delivery. For example, nanoparticles with targeted molecules can be designed with specific targeted molecules to achieve active targeting delivery using the targeted molecules to bind to receptors on the surface of tumor cells. At the same time, the size and surface properties of nanoparticles can be used to enable passive targeting accumulation in tumor tissue. The combination of these strategies can enhance the targeted delivery and therapeutic effectiveness of drugs. Overall, the combined effects of active and passive targeting can synergistically work to an advantage, overcoming the limitations of a single strategy to achieve more precise and effective drug delivery. Such combined strategies can help improve the effectiveness of oncology treatment and offer new possibilities for personalized therapy [125].

## 5. Future Perspectives and Conclusions

In recent years, research on nanodrugs has become more and more extensive and in-depth, due to the fact that nanoparticles have shown excellent anti-tumor effects on different types of tumors. Targeted drug delivery has an increasingly promising future in oncotherapy, and nanocarrier-based targeted drug delivery strategies are diverse due to different delivery methods and carriers. In this review, we mainly describe both passive and active targeting delivery strategies and systematically explain their advantages and disadvantages, respectively. Passive targeting facilitates the accumulation of nanoparticles accumulated into broad-spectrum tumors only through the physiological properties of tumor tissue that are different from normal healthy tissue, thereby achieving the purpose of enhancing anti-tumor effects and weakening toxic side effects of drugs.

Moreover, active targeting achieves precise drug delivery to specific tumors through highly specific binding between ligands and receptors, thus precisely delivering drugs directly to the targeted tumor cells and significantly improving the therapeutic effect. Both passive and active targeting strategies have their limitations and advancements in oncotherapy. A single treatment strategy is no longer the optimal solution, combined treatment is the best way to achieve a synergistic effect of ‘1 + 1 > 2’. Therefore, passive targeting and active targeting are synergized to work together on the disease site, ‘taking the best of both and discarding the worst’ to amplify the advantages of both and weaken the disadvantages, thus the treatment effect can be more efficient. For instance, magnetic field systems enable magnetic nanocarriers to penetrate deeper into the tumor, resulting in a several-fold increase in tumor accumulation. Combining active targeting with magnetic targeted optimizes the accumulation of nanoparticles at the tumor site and enhances therapeutic efficacy. Certainly, it is crucial to optimize the delivery system, but the response at the tumor site is needed. The internalization process of the nanocomplexes when it reaches the tumor site cannot be ignored, and a more in-depth exploration of the properties of the tumor itself and the mechanism of internalization is necessary. This approach will provide valuable insights into the mechanism of nanoparticle-based targeted drug delivery at the tumor site and greatly promote the clinical application of nanomedicines.

Recently, the strategies for loading drugs into nanoparticles in further targeted delivery will see the following developments in the future. Diversification of targeted molecules: Future research will aim to discover more molecules with specific binding to tumor cells such as more specific antibodies, peptides, and aptamers to achieve more accurate targeted effects. The innovative design of delivery vehicles, and new delivery vehicles will continue to be developed and optimized, offering improved stability, efficient drug loading capacity, and targeted release characteristics. The development of multimodal delivery systems, targeted DDS, will not be limited to a single delivery method but will combine different delivery strategies in an integrated manner to achieve more precise delivery and higher therapeutic efficacy. The personalized therapy is driven by the advancement of precision medicine and genetic testing, and molecular diagnostic techniques are employed to select the most suitable targeted drugs and delivery systems for patients, thereby enhancing therapeutic outcomes and reduces side effects. Collaboration between targeted immunotherapy, the combination of targeted drug delivery, and immunotherapy is becoming mainstream in oncotherapy. Combining immunotherapeutic agents with targeted molecules and delivery systems enables the activation and regulation of tumor immunity and improves the effectiveness of immunotherapy. However, the delivery system can be affected by the biological environment in the body such as the enzyme degradation and the adsorption of proteins in the blood circulation, which can lead to the failure of the delivery system or the occurrence of side effects. In addition, it is also a challenge to achieve clinical translation, large-scale production, and commercialization of delivery systems, which involves issues like the manufacturing process and the cost-effectiveness of the delivery system. That requires clinical testing and large-scale data analysis to validate the effectiveness and safety of targeted drug delivery in clinical applications. In summary, targeted drug delivery technology is a promising treatment tool that can offer patients more personalized, efficient, and safe tumor treatment options. However, this technology is still in a phase of continuous development and improvement, and further research and clinical trials are necessary to demonstrate its effectiveness and safety.

In recent years, a diverse array of anticancer nanomedicines has demonstrated impressive results in preclinical studies, enhancing therapeutic efficacy, minimize side effects, and increase survival rates [126,127,128]. However, only a select few nanomedicines have successfully transitioned from the bench to the bedside for clinical application. Presently, the predominant route of administration for nanomedicines is intravenous injection, utilizing either passive targeting or active targeting strategies to accumulate at the tumor site. As far as our knowledge extends, all nanomedicines approved by the U.S. FDA for cancer therapy rely on passive targeting to achieve tumor localization. Liposomes and polymeric nanoparticles are two types of nanomedicines that are typically approved for clinical intravenous delivery. For instance, DOX’s liposomal formulation, approved by the FDA as Doxil in 1995 and by the European Medicine Agency in 1996 as Caelyx, marked the first approved nanomedicines. Doxil reduces side effects compared to free adriamycin as it accumulates less in the heart [129]. Additionally, other anticancer nanomedicines utilizing passive targeting have also been clinically employed, including Paclical DHP107, Nanoxel, Onivyde, Marqibo, Abraxane, and ABI-009 [130]. Nonetheless, the EPR effect alone cannot fully meet the requirements for robust and effective drug delivery due to tumor heterogeneity, disease stage, and inter-patient variability [44,131]. As a result, only a limited number of active targeting-mediated nanomedicines have progressed to the clinical stage, including BIND-014, REXIN-G, and 2B3-101, all of which have successfully completed phase I/II trials [131,132]. However, there have been several actively targeted nanomedicines undergoing clinical trials that were terminated during phase I/II due to toxicity or other reasons such as MCC-465 and CALAA-01 [133,134]. Consequently, the clinical translation of nanomedicines remains challenging, and we firmly believe that two major hurdles must be overcome to achieve successful bench-to-bedside translation: (1) rational design of nanomedicines and strict quality control: thoughtful design of nanomedicines will bestow nanoparticles with optimal particle size, surface morphology, drug loading, charge, and drug release, along with other vital quality characteristics. This enhancement will further augment their in vivo properties, including biodistribution, controlled release, attenuation effect, receptor recognition, and more. As a result, nanomedicines will be better equipped to evade the immune system, penetrate tumors, target specific cells, and release drugs in a controlled manner. (2) Individualized nanomedicines as a driving force for the translation of actively targeted nanomedicines: cancer cell receptor expression is subject to complex interactions involving various factors such as organ, age, gender, race, environment, and more. Personalized therapy incorporates patient genomics, proteomics, and metabolomics. If personalized nanomedicines with active targeting properties are employed in cancer treatment, the expression of the patient’s cancer cell receptor genes and proteins, as well as the screening of metabolizing enzymes for targeted ligands that are not easily degraded, will collectively determine the success or failure of the individualized treatment approach. Addressing these challenges will pave the way for the successful clinical translation of nanomedicines, ensuring their potential to revolutionize cancer treatment and provide benefits to a diverse range of patients. On the contrary, by acquiring precise targeted receptors and metabolizing enzyme characteristics of each individual, and employing a rational design approach to create targeted receptor-specific ligands and ingeniously designing nanomedicines through the ‘gold standard’ of gene-protein–metabolism intertwinement, it becomes feasible to address the disparity between the vast number of preclinical studies and the limited number of anticancer nanomedicines that undergo true transformation.

We firmly believe that the rapid advancement of multidisciplinary technology, more nanomedicines with high efficacy, strong targeted capabilities, and excellent safety profiles will rapidly transition to clinical application, providing invaluable assistance to both doctors and patients. Moreover, the application of precision medicine will further facilitate the clinical transformation of targeted nanomedicines, addressing the limitations of conventional anticancer nanomedicines and delivering positive outcomes to the majority of patients. 

## Data Availability

Not applicable.

## Abbreviations

ADC	antibody-drug couples
ADCC	antibody-dependent cell-mediated cytotoxic effects
ADCP	antibody-dependent cytophagy
ALPPL2	alkaline phosphatase placenta-like 2
C5	component 5
CAF	cancer-associated fibroblasts
CCKBR	cholecystokinin B receptor
CDC	cytotoxic effects
CRC	colorectal cancer
CRP	complement regulatory protein
DC	dendritic cells
DDS	drug delivery systems
DLPC	1,2-Dilauroyl-sn-glycero-3-phosphorylcholine
DMPC	1,2-dimyristoyl-sn-glycero-3-phosphocholine
DOCP	2-((2,3-Bis(oleoyloxy)propyl)dimethylammonio)ethyl hydrogen Phosphate
DOCPe	2-((2,3-bis(oleoyloxy)propyl)-dimethylammonio)ethyl ethyl phosphate
DOX	doxorubicin
DPPC	1,2-Dipalmitoyl-sn-glycero-3-phosphocholine
DSPE-mPEG	1,2-distearoyl-sn-glycero-3-phosphoethanolamine with conjugated methoxyl PEG
ECM	extracellular matrix
EGFR	growth factor receptor
EPR	enhanced permeability and retention
ES	Estrone
FA-PEG	folic acid and poly(ethylene glycol)
FGNP	core–shell Fe_3_O_4_/Gd_2_O_3_ heterogeneous nanoparticles
GD2	glycosides deaminate
GEM	Gemcitabine
GM-CSF	granulocyte-macrophage colony stimulating factor
H_2_O_2_	hydrogen peroxide
ICG	indocyanine green
LPO	lipid peroxides
NE	nuclear envelope
NK	natural killer cell
NL	NGR peptide
NLS	nuclear targeting molecules
NPC	nuclear pore complex
NSCLC	non-small cell lung cancer
mAb	monoclonal antibody
MAC	membrane attack complex
mtDNA	mitochondrial DNA
MRI	magnetic resonance imaging
PAC1	phosphatase 1
PAMAM	Polyamidoamine
PBA	phenylboronic acid
PC	Phosphatidylcholine
PDAC	pancreatic ductal adenocarcinoma
PD-L1	programmed death ligand 1
PEG	polyethylene glycol
PET	positron emission tomography
PRR	pattern recognition receptor
PTX	paclitaxel-loaded
Que	quercetin
RGD	arginine–glycine–aspartate
ROS	reactive oxygen species
RPM	RGD modified paclitaxel (PTX)
SELEX	Systematic Evolution of Ligands by Exponential Enrichment
SFN	sorafenib
SPC	soy phosphatidylcholine
STCT	Subcellular targeting cancer therapy
TAA	tumor-associated antigens
TfR-BP	transferrin receptor binding peptide
TGF-α	Transforming growth factor α
TME	tumor microenvironment
TNBC	triple-negative breast cancer
TNF	tumor necrosis factor
TRC105	a monoclonal antibody against CD105
TSA	tumor-specific antigens
VEGF	vascular endothelial growth factor
ZH-1	a DNA aptamer
ZnO	zinc oxide
